# Supplementary material for: National Trends and Disparities in Complementary Food Diversity Among Infants: A 12-Year Cross-Sectional Birth Cohort Study
Source: Nutrients. 2025 Feb 11;17(4):636. doi: 10.3390/nu17040636 (PMC11858465; doi:10.3390/nu17040636)

Supplementary Table 1. Prevalence of the number of complementary food items introduced between 2009 and 2020 in the total population

Year	2009	2010	2011	2012	2013	2014	2015	2016	2017	2018	2,019	2020	*P* for trend
**Number of complementary food items**	**Number of participants, n (%)**												
6	60,428 (30.8)	71,050 (30.9)	82,490 (31.0)	86,006 (30.6)	102,424 (31.5)	105,111 (33.8)	138,457 (43.0)	165,827 (48.5)	144,578 (49.3)	146,215 (50.0)	138,552 (50.1)	139,873 (52.9)	<0.001
5	47,354 (24.2)	56,093 (24.4)	65,825 (24.7)	72,154 (25.6)	86,176 (26.5)	84,178 (27.1)	81,235 (25.3)	80,467 (23.5)	68,269 (23.3)	70,306 (24.1)	69,691 (25.2)	64,722 (24.5)	0.288
4	45,876 (23.4)	52,870 (23.0)	60,838 (22.9)	64,542 (22.9)	73,467 (22.6)	66,588 (21.4)	55,817 (17.4)	51,391 (15.0)	43,020 (14.7)	41,209 (14.1)	36,941 (13.3)	31,658 (12.0)	0.034
3	26,985 (13.8)	31,876 (13.9)	36,905 (13.9)	40,523 (14.4)	44,617 (13.7)	38,767 (12.5)	26,937 (8.4)	19,859 (5.8)	16,741 (5.7)	16,586 (5.7)	15,361 (5.5)	13,341 (5.0)	0.006
2	7,431 (3.8)	9,081 (4.0)	10,419 (3.9)	12,349 (4.4)	13,447 (4.1)	11,534 (3.7)	9,926 (3.1)	10,174 (3.0)	8,630 (2.9)	7,749 (2.7)	7,241 (2.6)	6,154 (2.3)	0.140
1	4,442 (2.3)	5,456 (2.4)	6,071 (2.3)	5,628 (2.0)	5,466 (1.7)	4,431 (1.4)	9,293 (2.9)	14,038 (4.1)	11,882 (4.1)	10,177 (3.5)	8,980 (3.2)	7,952 (3.0)	0.021
0	3,437 (1.8)	3,321 (1.4)	3,422 (1.3)	125 (0.04)	67 (0.02)	76 (0.02)	41 (0.01)	12 (0.0)	11 (0.0)	9 (0.0)	14 (0.01)	816 (0.31)	0.011

Supplementary Table 2. Prevalence of introduced complementary food items between 2009 and 2020 in the total population

Complementary food items (%)	2009	2010	2011	2012	2013	2014	2015	2016	2017	2018	2019	2020
Grains	88.2	87.9	85.2	86.7	87.0	87.9	88.2	88.9	89.1	89.7	90.0	89.8
Vegetables	93.6	95.6	92.7	96.5	97.0	97.8	94.7	92.3	92.9	93.3	93.6	93.6
Fruits	73.1	70.7	68.3	69	68.6	70.0	82.1	92.2	91.9	91.6	91.2	90.9
Eggs	53.6	54.1	53.1	55.8	58.3	60.0	64.5	65.8	62.0	65.2	64.2	66.6
Fish	53.9	54.2	52.8	54.6	55.6	59.3	64.8	65.5	68.4	70.0	72.5	75.7
Meats	88.2	90.2	88.9	93.0	94.2	95.5	93.3	91.7	92.3	92.9	93.3	93.7

Supplementary Figure 1. Flowchart of the study population

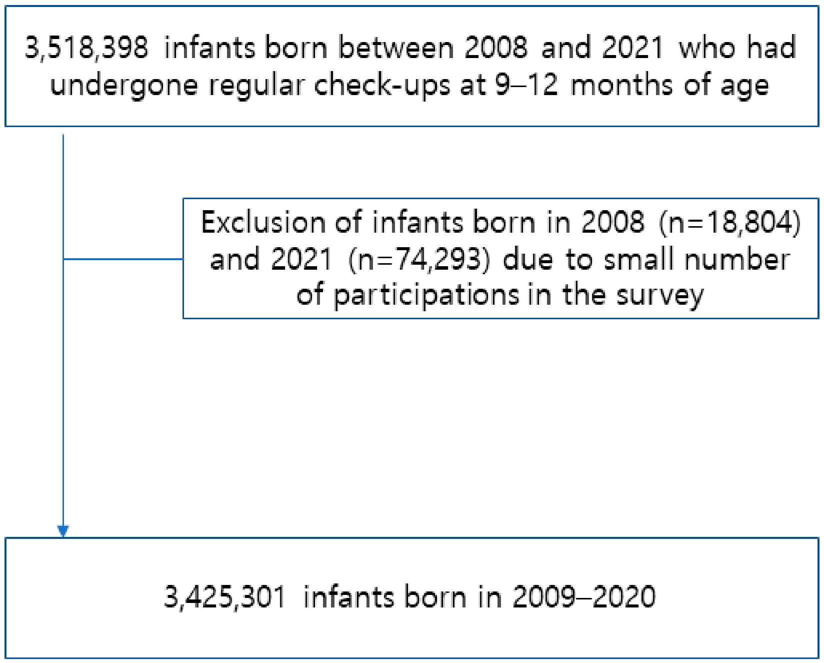

Supplement: Supplementary file 1 [file nutrients-17-00636-s001.zip › nutrients-3446663-supplementary.pdf]
